# The language-related transcription factor FOXP2 is post-translationally modified with small ubiquitin-like modifiers

**DOI:** 10.1038/srep20911

**Published:** 2016-02-12

**Authors:** Sara B. Estruch, Sarah A. Graham, Pelagia Deriziotis, Simon E. Fisher

**Affiliations:** 1 Language and Genetics Department, Max Planck Institute for Psycholinguistics, Wundtlaan 1, 6525 XD, Nijmegen, The Netherlands; 2 Donders Institute for Brain, Cognition and Behaviour, Radboud University, 6525 EN, Nijmegen, The Netherlands

## Abstract

Mutations affecting the transcription factor FOXP2 cause a rare form of severe speech and language disorder. Although it is clear that sufficient FOXP2 expression is crucial for normal brain development, little is known about how this transcription factor is regulated. To investigate post-translational mechanisms for FOXP2 regulation, we searched for protein interaction partners of FOXP2, and identified members of the PIAS family as novel FOXP2 interactors. PIAS proteins mediate post-translational modification of a range of target proteins with small ubiquitin-like modifiers (SUMOs). We found that FOXP2 can be modified with all three human SUMO proteins and that PIAS1 promotes this process. An aetiological FOXP2 mutation found in a family with speech and language disorder markedly reduced FOXP2 SUMOylation. We demonstrate that FOXP2 is SUMOylated at a single major site, which is conserved in all FOXP2 vertebrate orthologues and in the paralogues FOXP1 and FOXP4. Abolishing this site did not lead to detectable changes in FOXP2 subcellular localization, stability, dimerization or transcriptional repression in cellular assays, but the conservation of this site suggests a potential role for SUMOylation in regulating FOXP2 activity *in vivo*.

Heterozygous disruption of the *FOXP2* gene, which encodes a member of the forkhead box (FOX) family of transcription factors, leads to a rare and severe form of speech and language disorder (MIM 605317 (gene), 602081 (disorder)). This developmental disorder was first described in a three-generation pedigree (the KE family), in which half of the family members have difficulties with learning to make co-ordinated orofacial movements underlying speech (childhood apraxia of speech), together with wide-ranging impairments in comprehension and production of spoken and written language, but without serious impact on other aspects of cognitive functioning^1^. All affected members of the KE family carry a missense mutation (R553H) within the FOX DNA-binding domain, which abolishes DNA binding and transcriptional repression by FOXP2^1^,^2^,^3^. Around twenty further cases of speech/language disorder resulting from *FOXP2* haploinsufficiency have since been reported, including nonsense and frameshift point mutations, as well as chromosomal rearrangements disturbing the locus^4^,^5^,^6^,^7^. Thus, adequate FOXP2 expression appears to be essential for normal development of language-related brain circuits, presumably in order to establish correct expression levels of crucial downstream target genes involved in processes such as neurite outgrowth and synaptic plasticity^8^,^9^.

*FOXP2* shows evolutionarily-conserved expression in brain structures including the cortex, basal ganglia, thalamus and cerebellum^10^,^11^. Studies in animal models further support the notion that precisely controlled levels of FOXP2 are necessary for normal brain development (as reviewed in^12^,^13^). Mice in which both copies of the *Foxp2* gene have been disrupted show severe motor impairments and developmental delay, and die 3–4 weeks after birth^9^,^13^ (Note that the murine orthologue of *FOXP2* is designated as *Foxp2* and orthologues in other species as *FoxP2*). When mice are heterozygous for a *Foxp2* mutation equivalent to that found in the KE family, they are overtly normal, but exhibit deficits in motor skill learning and abnormal electrophysiology within cortico-striatal circuits^9^,^13^,^14^. Transient manipulation of *Foxp2* levels also has deleterious consequences in the developing mouse brain: both knock-down and overexpression of *Foxp2* have been reported to affect neurogenesis, neuronal morphology and migration^15^,^16^. The effect of manipulating *FoxP2* levels has also been investigated in the zebra finch, a species which, like humans, has the unusual ability to learn vocalizations from other individuals^12^. Both knock-down and overexpression of *FoxP2* in key parts of the brains of juvenile zebra finches disrupt the normal process of song learning^17^,^18^, indicating that precise control of FoxP2 levels is necessary for normal vocal learning behaviour in this species.

Studies in songbirds indicate that, in addition to the spatial regulation of expression, dynamic temporal regulation of activity of this transcription factor is important for its functions in the developing and adult brain. Zebra finch *FoxP2* expression is elevated in a specific song-related brain region (Area X) during the critical period in which juvenile birds learn their song^19^,^20^. Furthermore, in adult birds, *FoxP2* levels in Area X decrease when males practice songs alone, but not during performance of songs to females, which may contribute to the increased variability in song output during solo practice compared to female-directed singing^21^,^22^,^23^,^24^. Similar dynamic regulation of the human orthologue could potentially play a role in vocal learning during speech acquisition.

While several studies have examined patterns of FOXP2 protein expression in the brains of different species^10^,^11^,^25^, few investigations have addressed potential mechanisms for regulation of FOXP2 activity^26^. Transcription factor activity is often regulated via interaction with other transcription factors, co-repressors/co-activators, chromatin-modifiers, and post-translational modification enzymes. Such interactions can alter protein turnover, increase or decrease transcriptional activation/repression activity, or influence selection of downstream targets. A small number of FOXP2-interacting proteins have been described, notably the paralogues FOXP1 and FOXP4, the transcription factor TBR1, and the co-repressor CtBP1^27^,^28^,^29^,^30^. To uncover additional mechanisms for regulation of FOXP2 activity we sought to identify novel protein interaction partners. We found that members of the PIAS family of proteins interact with FOXP2, and also with the paralogue FOXP1. PIAS proteins mediate post-translational modification of nuclear proteins with small ubiquitin-like modifiers (SUMOs)^31^,^32^. SUMOs are ubiquitously-expressed polypeptides that are reversibly coupled to many different proteins with a variety of functional outcomes. We show that FOXP2 is modified with SUMOs at a single major evolutionarily-conserved site, and that PIAS1 promotes this modification. SUMOylation of FOXP2 is an excellent candidate mechanism for dynamic regulation of FOXP2 activity *in vivo*.

## Results

### FOXP2 interacts with members of the PIAS family of proteins

**Figure 1:**
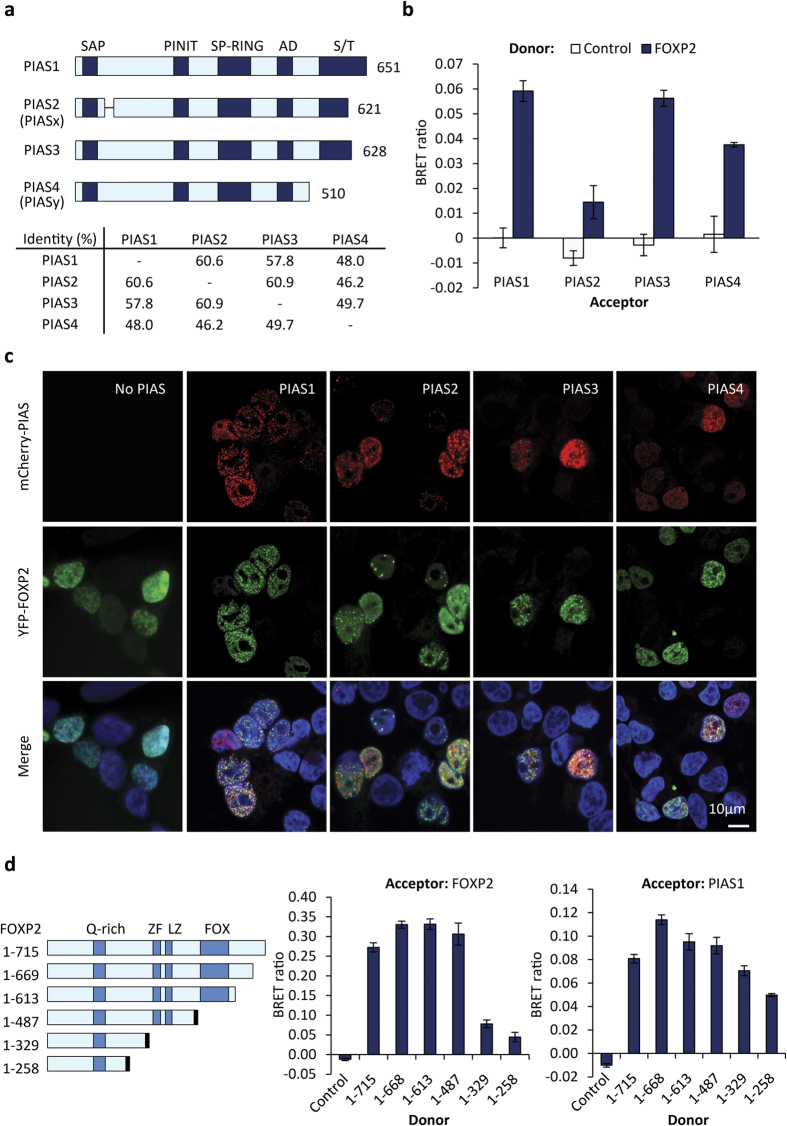
FOXP2 interacts with members of the PIAS family of SUMO E3 ligases. (**a**) Top: schematic representation of human PIAS proteins. Domains are shaded in dark blue: SAP domain (SAP); PINIT domain (PINIT); SP-RING domain (SP-RING); acidic domain (AD); serine/threonine-rich domain (S/T). The number of amino acid residues is shown to the right of the schematic. Bottom: identity matrix for PIAS proteins. (**b**) BRET assay for interaction between FOXP2 and PIAS proteins. HEK293 cells were transfected with luciferase-FOXP2 (donor) and YFP-PIAS (acceptor). The control donor protein is a nuclear-targeted luciferase. Values are mean corrected BRET ratios ± S.E.M. (n = 3). (**c**) Fluorescence micrographs of HEK293 cells transfected with mCherry-PIAS (red) and YFP-FOXP2 (green). Nuclei were stained with Hoechst 33342 (blue). (**d**) Left panel: Schematic representation of synthetic truncated forms of FOXP2. The number of amino acid residues is shown on the left; 1–715 represents the full-length protein. Known domains are shown in dark blue: glutamine-rich region (Q-rich); zinc finger (ZF); leucine zipper (LZ); forkhead domain (FOX). A nuclear-targeting signal (shown in black) was appended to the C-terminus of variants 1–487, 1–329, and 1–258 because these variants lack endogenous nuclear targeting signals. Centre panel: BRET assay for interaction between synthetic FOXP2 truncations and wild-type FOXP2. HEK293 cells were transfected with luciferase-FOXP2 truncations (donor) and YFP-FOXP2 (acceptor) Right panel: BRET assay for the interaction between synthetic FOXP2 truncations and PIAS1. HEK293 cells were transfected with luciferase-FOXP2 truncations (donor) and YFP-PIAS1 (acceptor). The control donor protein is a nuclear-targeted luciferase. Values are mean corrected BRET ratios ± S.E.M. (n = 3).

To identify candidate interaction partners of FOXP2, a screen of a human foetal brain yeast two-hybrid library was conducted using the full-length human protein as bait. The most frequently observed prey in this screen was PIAS1 (Supplementary Table S1). The vertebrate PIAS family includes four proteins with conserved domain architecture and 45–60% sequence identity (Fig. 1a). Interestingly, PIAS3 was one of four proteins identified as candidate FOXP2 interactors in an independent screen of a human foetal brain yeast two-hybrid library, also using full-length human FOXP2 as the bait^33^. PIAS proteins are known to interact with and modulate the activity of a range of transcription factors^32^,^34^. Members of the PIAS family therefore appeared to be strong candidates for FOXP2 interaction partners.

In order to confirm the interaction of FOXP2 with PIAS proteins, we used a Bioluminescence Resonance Energy Transfer (BRET) assay, which allows protein-protein interactions to be monitored in live mammalian cells in culture^29^. In the BRET assay, a protein of interest is expressed as a fusion with *Renilla* luciferase (Luc) and a candidate interaction partner is expressed as a fusion with yellow fluorescent protein (YFP). An interaction between the two proteins brings the Luc and YFP moieties into sufficient proximity to allow resonance energy transfer to occur upon addition of a luciferase substrate, shifting the wavelength of the emitted light from 480 nm to 530 nm. Using Luc-FOXP2 and YFP-PIAS fusion proteins, we confirmed that FOXP2 interacts with PIAS1 and PIAS3, and in addition demonstrated interaction with PIAS4, and a potential weaker interaction with PIAS2 (Fig. 1b). Note that all experiments were performed using HEK293 cells, unless indicated otherwise.

We noted that PIAS proteins exhibited nuclear localization with a distinctive speckled appearance, as has been reported previously^34^. We therefore examined if co-expression of PIASs with FOXP2 would cause redistribution of FOXP2, which normally exhibits a diffuse localization within nuclei. Expression of PIAS1 together with FOXP2 caused a dramatic change, involving extensive co-localization of FOXP2 with PIAS1 in nuclear speckles (Fig. 1c). A similar effect was observed upon expression of PIAS3 and PIAS4, consistent with the interaction observed in the BRET assay. Upon overexpression of PIAS2, FOXP2 retained a largely diffuse nuclear distribution, with little FOXP2 exhibiting co-localization with PIAS2 within speckles, consistent with the lower level of interaction observed between FOXP2 and PIAS2 in BRET experiments. The interaction between FOXP2 and PIASs may therefore draw FOXP2 into nuclear speckles.

To try to identify the region of FOXP2 involved in binding to PIAS proteins, we performed BRET assays using a series of synthetic, truncated versions of FOXP2^28^ (Fig. 1d). These truncations appear to be effective in mapping interaction sites because deletion of the region containing residues 330–487, which contains the leucine zipper dimerization domain^30^, results in a substantial reduction in interaction with full-length FOXP2 (Fig. 1d, centre). Notably, even the shortest FOXP2 truncation tested (residues 1–258) retained the ability to interact with PIAS1, though perhaps to a slightly lesser degree than the full-length protein (Fig. 1d, right). These results suggest that some key determinants of PIAS binding reside within the N-terminal region of FOXP2. Apart from a polyglutamine tract, this region does not contain any known domains, but it does include regions of polypeptide that are highly conserved in FOXP1 and FOXP4, and has also been identified as the region interacting with the autism-related transcription factor TBR1, suggesting that this region may coordinate multiple protein-protein interactions^28^.

### FOXP2 is SUMOylated

**Figure 2:**
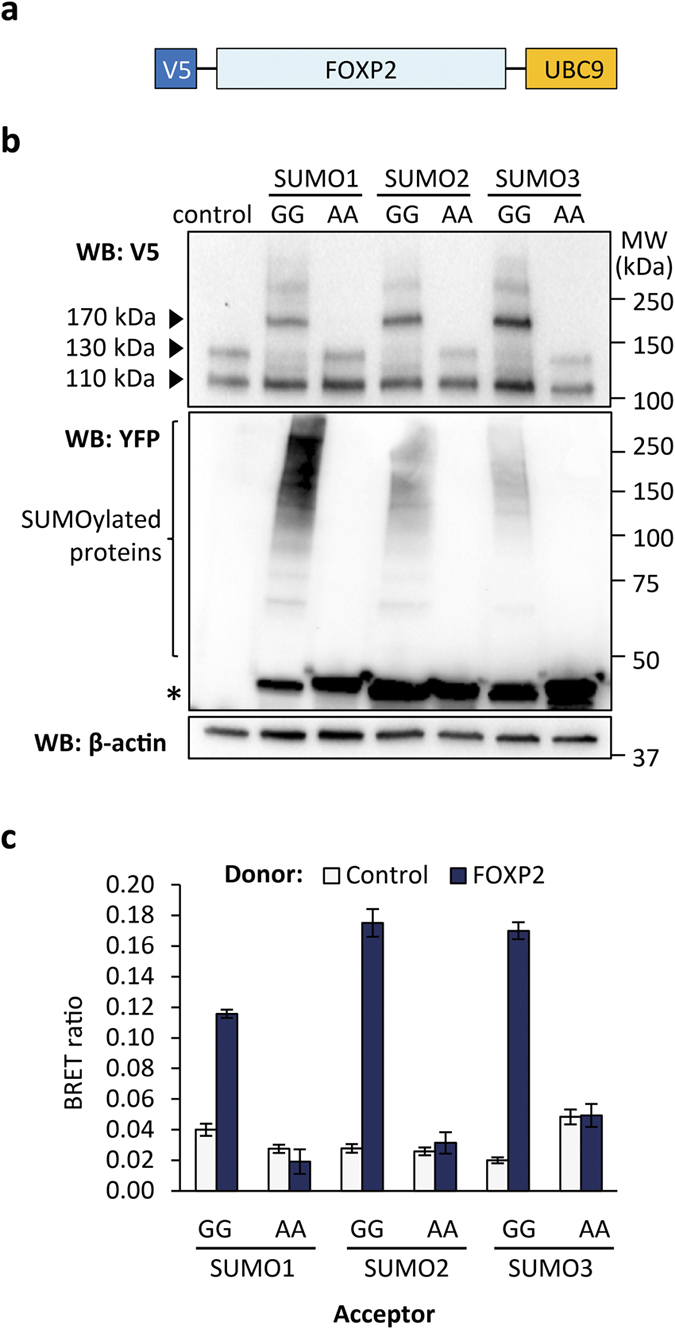
FOXP2 can be SUMOylated with SUMO1, 2 and 3. (**a**) Schematic representation of the FOXP2-UBC9 fusion protein with an N-terminal V5 epitope tag. (**b**) Gel shift assay for FOXP2 SUMOylation. FOXP2-UBC9 was expressed in HEK293 cells together with a YFP-fusion of either wild-type SUMO (GG), or mutant SUMO in which the two C-terminal glycine residues required for conjugation to the target protein were mutated to alanine (AA), or with YFP alone (control). Top panel: western blot probed with anti-V5 antibody to detect FOXP2-UBC9. The 110 kDa species is unmodified FOXP2-UBC9. The 130 kDa species is FOXP2-UBC9 modified with endogenous SUMO. The 170 kDa species is FOXP2-UBC9 modified with YFP-SUMO. Middle panel: western blot probed with anti-YFP antibody. The asterisk indicates unconjugated YFP-SUMO. Higher molecular weight species are cellular proteins modified with YFP-SUMO. Bottom panel: western blot probed with anti-β-actin to confirm equal loading. (**c**) BRET assay for interaction between FOXP2 and SUMO. HEK293 cells were transfected with luciferase-FOXP2 (donor) and YFP-SUMO (acceptor), using either wild-type SUMO (GG) or alanine mutants (AA). The control donor protein is a nuclear-targeted luciferase. Values are mean corrected BRET ratios ± S.E.M. (n = 3).

PIAS proteins function as SUMO E3 ligases, promoting the transfer of SUMO from the SUMO-conjugating enzyme UBC9 to an acceptor lysine residue in a target protein, in a manner analogous to the transfer of ubiquitin to proteins by ubiquitin E3 ligases^31^,^32^. There are three SUMO proteins in vertebrates, SUMO1, SUMO2 and SUMO3, all of which have a molecular weight of 11 kDa. SUMO2 and SUMO3 have ~95% amino acid sequence identity and are thought to be functionally very similar, whereas SUMO1 has only ~50% amino acid sequence identity with SUMO2/3 and is not functionally redundant with these proteins^31^. The SUMOylation of specific proteins is typically difficult to detect due to the dynamic nature of the modification, which is readily removed by SUMO-specific proteases of the SENP family, and the fact that only a minor proportion of target protein molecules carry a SUMO moiety at any one time^31^. To facilitate detection of protein SUMOylation, the 18 kDa SUMO-conjugating enzyme UBC9 can be fused to a target protein of interest^35^. We therefore generated a FOXP2-UBC9 fusion construct, which also carries a V5 epitope tag to enable the fusion to be detected independently from endogenous FOXP2 (Fig. 2a).

We transfected cells with FOXP2-UBC9 together with a YFP-fusion of SUMO1, SUMO2 or SUMO3. The use of YFP-tagged SUMOs allows discrimination between proteins modified with endogenous and exogenous SUMO. Lysates of transfected cells were probed by western blotting with anti-V5 antibody to detect any shift in the migration of FOXP2-UBC9 resulting from SUMOylation (Fig. 2b). All samples contained a FOXP2-UBC9 species that migrated at ~110 kDa, representing unSUMOylated protein. Cells transfected with FOXP2-UBC9 and a YFP-SUMO contained a ~170 kDa FOXP2-UBC9 species that was not present in control cells transfected with FOXP2-UBC9 and YFP, indicating that FOXP2 can be SUMOylated with all three SUMOs (Fig. 2b). To confirm that the ~170 kDa species represents YFP-SUMO conjugated to FOXP2-UBC9, we generated mutant forms of YFP-SUMO, in which the two C-terminal glycine residues required for conjugation to target proteins (and to UBC9) were mutated to alanine. As expected, cells transfected with FOXP2-UBC9 together with an alanine mutant YFP-SUMO did not contain the ~170 kDa species (Fig. 2b). In cells transfected with FOXP2-UBC9 together with a mutant YFP-SUMO or YFP alone, a ~130 kDa FOXP2-UBC9 species was observed, which may represent FOXP2-UBC9 modified with endogenous SUMO (Fig. 2b). Note that the observed molecular weights of the different FOXP2-UBC9 species do not necessarily correspond with theoretical values because SUMOylated proteins are branched polypeptides and exhibit anomalous migration.

In order to confirm the results of the gel shift assay, we also examined FOXP2 SUMOylation in a BRET assay using Luc-FOXP2 with YFP-SUMO fusion proteins. Interaction of FOXP2 was observed with SUMO1, SUMO2 and SUMO3, but not with their respective alanine mutants, in agreement with the gel shift assay (Fig. 2c). Notably, the BRET assay readily allows detection of FOXP2 SUMOylation without the need to fuse FOXP2 to UBC9, indicating that the technique is highly sensitive for monitoring SUMOylation. BRET has rarely been used in studies of SUMOylation, but may be a widely applicable technique for examining this modification, with the advantage that its use in live cells overcomes the difficulties in maintaining protein SUMOylation encountered in most experimental procedures.

### PIAS1 promotes SUMOylation of FOXP2

**Figure 3:**
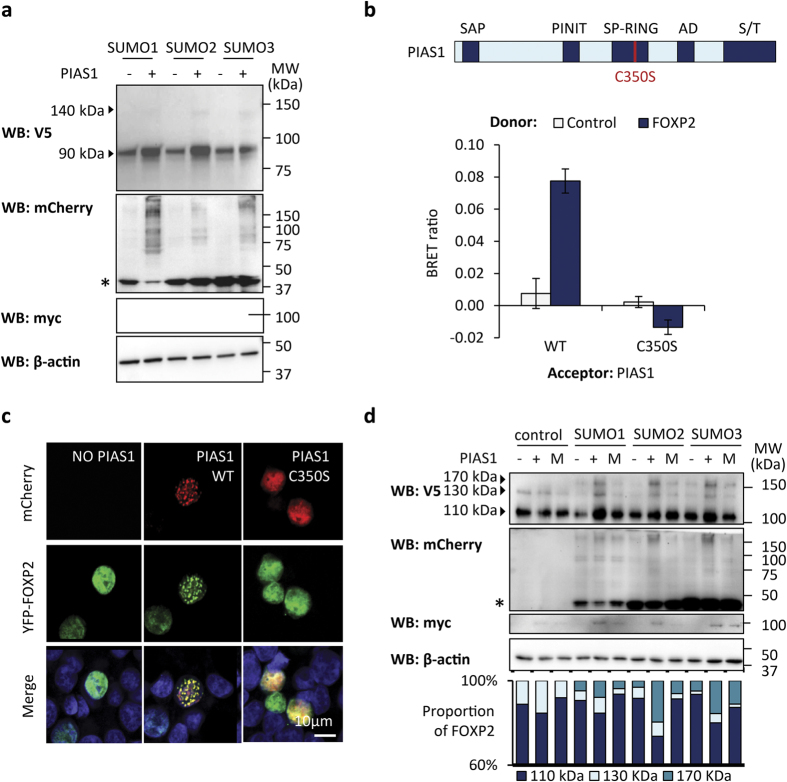
PIAS1 promotes FOXP2 SUMOylation. (**a**) Gel shift assay for SUMOylation of FOXP2. HEK293 cells were transfected with V5-tagged FOXP2 and mCherry-SUMO, together with myc-tagged PIAS1 (+) or an empty vector (−). Top panel: western blot probed with anti-V5 antibody. The 90 kDa species is unmodified FOXP2. The 140 kDa species is FOXP2 modified with mCherry-SUMO. Second panel: western blot probed with anti-mCherry. The asterisk indicates unconjugated mCherry-SUMO. Higher molecular weight species are cellular proteins modified with mCherry-SUMO. Third panel: western blot probed with anti-myc tag to detect PIAS1. Bottom panel: western blot probed with anti-β-actin to confirm equal loading. (**b**) Top: schematic representation of PIAS1 C350S mutant. Bottom: BRET assay for interaction of FOXP2 with PIAS1. HEK293 cells were transfected with luciferase-FOXP2 (donor) and YFP-PIAS1 (wild-type (WT) or C350S mutant, acceptor). The control donor protein is a nuclear-targeted luciferase. Values are mean corrected BRET ratios ± S.E.M. (n = 3). (**c**) Fluorescence micrographs of HEK293 cells transfected with mCherry-tagged wild-type PIAS1 (WT) or C350S mutant (red) and YFP-FOXP2 (green). Nuclei were stained with Hoechst 33342 (blue). (**d**) Gel shift assay for SUMOylation of FOXP2. HEK293 cells were transfected with FOXP2-UBC9 together with mCherry-SUMO or mCherry alone (control) and myc-tagged wild-type PIAS1 (+), C350S mutant (M) or empty vector (−). Top panel: western blot probed with anti-V5 antibody to detect FOXP2-UBC9. The 110 kDa species is unmodified FOXP2-UBC9. The 130 kDa species is FOXP2-UBC9 modified with endogenous SUMO. The 170 kDa species is FOXP2-UBC9 modified with mCherry-SUMO. Second panel: western blot probed with anti-mCherry antibody. The asterisk indicates unconjugated mCherry-SUMO. Higher molecular weight species are cellular proteins modified with mCherry-SUMO. Third panel: western blot probed with anti-myc to detect PIAS1. Fourth panel: western blot probed with anti-β-actin to confirm equal loading. Bottom panel: densitometry analysis of FOXP2-UBC9 species.

To determine if PIAS proteins are involved in the SUMOylation of FOXP2, we focused on PIAS1, the PIAS family member identified in the original yeast two-hybrid screen. We tested if overexpression of PIAS1, together with SUMO, would allow SUMOylation of FOXP2 to be detected in a gel shift assay without the need to fuse FOXP2 to UBC9. Overexpression of myc-tagged PIAS1 together with any of the three SUMO proteins (fused to mCherry) gave rise to a new FOXP2 species of ~140 kDa, suggesting that PIAS1 is able to stimulate SUMOylation of FOXP2 (Fig. 3a). Importantly, the observation of a new high molecular weight FOXP2 species in this experiment shows that FOXP2 can be SUMOylated without being fused to UBC9.

To confirm that the increase in SUMOylation was mediated directly by PIAS1, we generated a catalytically inactive version of PIAS1 that has a point mutation (C350S) within the SP-RING domain, which is involved in the recognition of target proteins (Fig. 3b)^32^,^36^. The C350S PIAS1 mutant was unable to interact with FOXP2 in a BRET assay, indicating that the SP-RING domain may be involved in recognition of FOXP2 as a SUMOylation target (Fig. 3b). The C350S mutant displayed a more diffuse nuclear localization than wild-type PIAS1, suggesting that the localization of PIAS1 within nuclear speckles is connected to its activity as a SUMO E3 ligase (Fig. 3c). Consistent with this, the C350S mutant did not induce redistribution of FOXP2 into nuclear speckles (Fig. 3c). In a gel shift assay, wild-type PIAS1 promoted the modification of FOXP2-UBC9 with both endogenous SUMO (~130 kDa species) and mCherry-tagged SUMO (~170 kDa species) (Fig. 3d). The C350S mutant was unable to promote SUMOylation of FOXP2-UBC9 (Fig. 3d), indicating that the increase in FOXP2 SUMOylation observed upon PIAS1 overexpression is due to the SUMO E3 ligase activity of PIAS1.

### K674 is the major SUMOylation site in FOXP2

**Figure 4:**
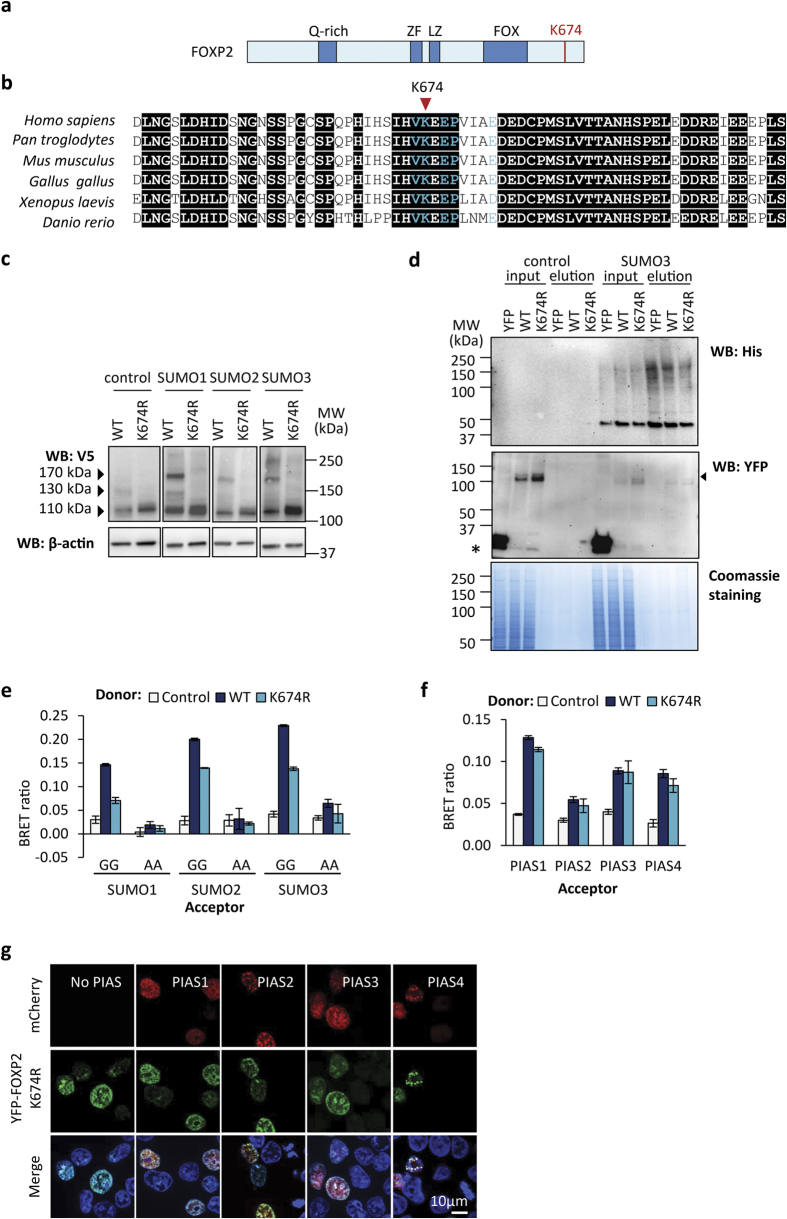
K674 is the major SUMOylation site in FOXP2. (**a**) Schematic representation of FOXP2 showing the predicted SUMOylation site. Known domains are shown in dark blue: glutamine-rich region (Q-rich); zinc finger (ZF); leucine zipper (LZ); forkhead domain (FOX). (**b**) Sequence alignment of the region surrounding the putative SUMOylation site in FOXP2 orthologues. Conserved residues are shown on a black background. Critical residues of the KEPE-type SUMOylation site motif are shown in turquoise. UniProt accession numbers: *Homo sapiens* O15409; *Pan troglodytes* Q8MJ98; *Mus musculus* P58463; *Gallus gallus* Q5IHK1; *Xenopus laevis* Q4VYS1; *Danio rerio* Q4JNX5. (**c**) Gel shift assay for FOXP2 SUMOylation. HEK293 cells were transfected with FOXP2-UBC9 (wild-type (WT) or K674R mutant) together with YFP-SUMOs or YFP alone (control). Top panel: western blot probed with anti-V5. The 110 kDa species is unmodified FOXP2-UBC9. The 130 kDa species is FOXP2-UBC9 modified with endogenous SUMO. The 170 kDa species is FOXP2-UBC9 modified with YFP-SUMO. Bottom panel: western blot probed with anti-β-actin. (**d**) Pull-down assay for FOXP2 SUMOylation. HeLa cells stably expressing His-tagged SUMO3, or the parental HeLa cell line (control), were transfected with YFP-FOXP2 (wild-type (WT) or K674R mutant) or YFP alone. His-tagged species were isolated under denaturing conditions using cobalt affinity purification. Western blots of total lysate (input) and affinity-purified material (elution) were probed with anti-His tag antibody to visualize SUMO-conjugated proteins (top panel). YFP and YFP-FOXP2 were visualized using anti-GFP antibody: FOXP2 is indicated by an arrowhead and YFP with an asterisk (middle panel). Total protein was visualized by Coomassie blue staining (bottom panel). (**e**) BRET assay for interaction of FOXP2 with SUMO. HEK293 cells were transfected with luciferase-FOXP2 (wild-type (WT) or K674R mutant, donor) and YFP-SUMO (wild-type (GG) or alanine mutant (AA), acceptor). The control is a nuclear-targeted luciferase. Values are mean corrected BRET ratios ± S.E.M. (n = 3). (**f**) BRET assay for interaction of FOXP2 and PIAS proteins. Cells were transfected with luciferase-FOXP2 (wild-type (WT) or K674R mutant, donor) and YFP-PIAS (acceptor). (**g**) Fluorescence micrographs of HEK293 cells transfected with mCherry-PIAS (red) and YFP-FOXP2 K674R mutant (green). Nuclei were stained with Hoechst 33342 (blue).

SUMOs are conjugated to target proteins via an isopeptide bond formed by the C-terminal carboxyl group of SUMO and the amino group of a lysine side chain in the target protein. Lysine residues that are subject to SUMOylation are often found within the consensus sequence ΨKX(D/E), where Ψ is a hydrophobic amino acid and X is any amino acid, although many SUMOylation sites do not conform to this pattern^37^. We used three prediction algorithms to identify potential SUMOylation sites in FOXP2: SUMOplot (www.abgent.com/sumoplot), GPS-SUMO (sumosp.biocuckoo.org)^38^, and JASSA (www.jassa.fr)^39^. All three algorithms identified K674 as a high-confidence potential SUMOylation site (Supplementary Table S2). No other lysine residues in FOXP2 were identified as potential SUMOylation sites by more than one algorithm. Residue K674 lies in the C-terminal region of FOXP2, which does not contain any previously described functional domains, consistent with the typical localization of SUMOylation sites within structurally disordered regions of polypeptide (Fig. 4a)^40^. This residue is within a VKEE sequence that matches the consensus ΨKX(D/E) motif (Fig. 4b). In addition, the putative SUMOylation site at K674 belongs to a class of predicted SUMOylation sites termed KEPE motifs, which have the consensus sequence ΨKX(D/E)PXXX(D/E)^41^. KEPE motifs are found in over 130 human proteins, and are enriched among proteins involved in transcription^41^. The critical residues of the KEPE motif are conserved in vertebrate FOXP2 proteins, supporting a functional role for the motif (Fig. 4b).

To assess if K674 functions as a SUMOylation site, we mutated this residue to arginine, thus removing the amino group required for SUMO conjugation but preserving the positive charge at this position of the polypeptide. We performed a gel shift assay using wild-type or mutant FOXP2-UBC9 co-transfected with PIAS1 and each of the three SUMOs. In this assay, the ~170 kDa SUMOylated FOXP2-UBC9 species observed in cells transfected with wild-type FOXP2 was not present in cells transfected with the K674R mutant (Fig. 4c). Thus K674 is the major site in FOXP2 that is subject to modification by SUMO1, SUMO2 and SUMO3.

The dramatic reduction in SUMOylation resulting from mutation of residue K674 suggests that this residue may be the most important SUMOylation site *in vivo*. To detect if a proportion of FOXP2 might be SUMOylated at alternative sites, we employed a HeLa cell line stably expressing His-tagged SUMO3^42^. Using cobalt affinity purification under denaturing conditions, SUMOylated species were purified from HeLa-SUMO3 cells transfected with YFP-tagged wild-type FOXP2, YFP-tagged K674R mutant, or YFP alone (Fig. 4d). YFP-FOXP2 was detected in the affinity-purified protein fraction from the HeLa-SUMO3 cells, confirming that FOXP2 can be SUMOylated without being fused to UBC9 (Fig. 4d). Note that in this key experiment we did not detect an observable shift in the molecular weight of FOXP2 as a result of SUMOylation, in contrast to the clear size shifts observed in our earlier experiments. One potential explanation of the discrepancy is that this experiment employed His-tagged SUMO, with a molecular weight of only ~10 kDa, comparable to that of endogenous SUMO, while our prior experiments used YFP- and mCherry-tagged SUMO proteins, which have substantially higher molecular weights of >35 kDa. Importantly, YFP-FOXP2 was not detectable among proteins eluted from resin incubated with lysate from the parental HeLa cell line, demonstrating that FOXP2 does not bind non-specifically to the affinity resin (Fig. 4d). Moreover, the YFP control protein was not detectable in the affinity-purified fraction from the HeLa-SUMO3 cells, demonstrating that only SUMOylated proteins are purified using this procedure (Fig. 4d).

The K674R mutant was also present in the affinity-purified material from the HeLa-SUMO3 cell line, indicating that specific enrichment of SUMOylated proteins allows detection of rarer forms of FOXP2 that are SUMOylated at one or more alternative sites (Fig. 4d). Different SUMOylation site prediction tools variously identify potential additional SUMOylation sites at K74, K285, K417 and K560 (Supplementary Table S2). However, none of these predictions are consistent across two or more prediction tools, and none lie within a typical consensus SUMOylation motif. While it is possible that several additional sites in FOXP2 may occasionally be SUMOylated, modification at these sites may not serve a critical biological function.

In a BRET assay, the K674R mutant displayed consistently reduced, but not abolished, interaction with wild-type SUMO1, SUMO2 and SUMO3, and no interaction with the respective alanine mutants (Fig. 4e). The residual interaction between the K674R mutant and SUMOs in the BRET assay might be accounted for by the presence of minor secondary SUMOylation sites. Given that the reduction in the BRET signal resulting from the K674R mutation is modest, there may also be a contribution to this signal from non-covalent association of the mutant with SUMO. Low-affinity, non-covalent interactions between SUMOs and other proteins are mediated by SUMO-interaction motifs (SIMs)^31^. We employed the JASSA^39^ and GPS-SUMO^38^ algorithms to identify potential SIMs in FOXP2, but no high-confidence SIMs were found (Supplementary Table S3).

SUMO may instead associate with the K674R mutant as part of a complex with UBC9 and PIAS. In support of this model, the K674R mutant exhibited a similar degree of interaction with PIASs as wild-type FOXP2 in a BRET assay (Fig. 4f). We also observed co-localization of the K674R mutant with PIASs in nuclear speckles (Fig. 4g). Therefore K674 is not required for interaction of FOXP2 with PIAS proteins, consistent with the mapping of the PIAS binding site to the N-terminal region of FOXP2 (Fig. 1d). Furthermore, the relocalization of FOXP2 to nuclear speckles that is observed upon overexpression of PIASs appears to be due to the interaction between PIAS and FOXP2, and not to the SUMOylation of FOXP2.

### Functional consequences of FOXP2 SUMOylation

**Figure 5:**
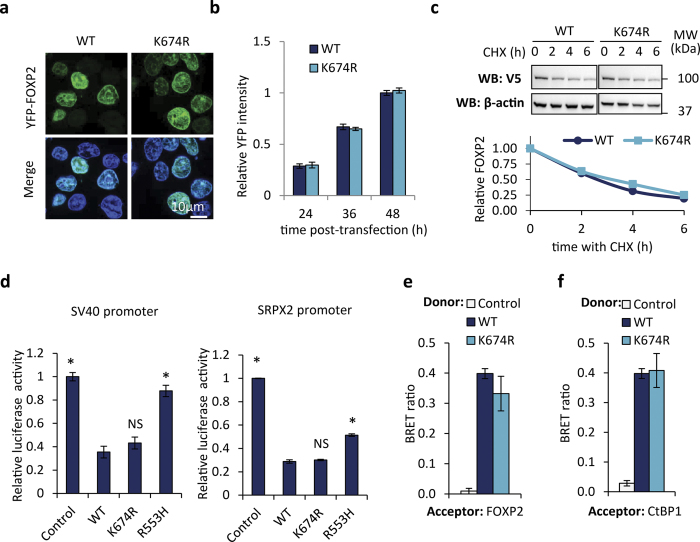
Abolishing the K674 SUMOylation site has no effect in cellular assays of FOXP2 function. (**a**) Fluorescence micrographs of cells transfected with YFP-tagged wild-type (WT) and K674R mutant forms of FOXP2. Nuclei were stained with Hoechst 33342 (blue). (**b**) Fluorescence-based measurement of FOXP2 expression level. HEK293 cells were transfected with YFP-FOXP2 (wild-type (WT) or K674R mutant), together with mCherry for normalization. Fluorescence intensity was measured 24, 36 and 48 h post-transfection. Values are mean YFP/mCherry fluorescence ratios ± S.E.M. (n = 3), relative to the value for wild-type FOXP2 at 48 h. (**c**) Western blot assay for FOXP2 degradation. HEK293 cells were transfected with V5-tagged FOXP2 (wild-type (WT) or K674R mutant). Cycloheximide (CHX) was added to cells 48 h after transfection for varying amounts of time. Top: western blots of whole cell extracts probed with anti-V5 and anti-β-actin antibodies. Bottom: densitometry quantification of FOXP2. Values are normalized to β-actin and plotted relative to the 0 h time point. (**d**) Luciferase reporter assays for transcriptional regulatory activity of FOXP2. HEK293 cells were transfected with a luciferase reporter vector containing the SV40 promoter (left) or the human SRPX2 promoter (right), together with YFP-FOXP2 (wild-type (WT), K674R or R553H mutants), or YFP alone (control). Values are mean relative luciferase activity ± S.E.M. (n = 3), expressed relative to the control. Asterisks indicate significant differences compared to wild-type FOXP2 (p < 0.05, one-tailed student’s t-test). NS, not significant. Exact p-values for the SV40 assay are 0.0043 for the control, 0.0048 for R553H, 0.1598 for K674R. Exact p-values for the SRPX2 assay are 0.0009 for the control, 0.0017 for R553H, and 0.2566 for K674R. (**e**) BRET assay for FOXP2 dimerization. HEK293 cells were transfected with luciferase-FOXP2 (wild-type (WT) or K674R mutant, donor) and YFP-FOXP2 (acceptor). The control is a nuclear-targeted luciferase. Values are mean corrected BRET ratios ± S.E.M. (n = 3). (**f**) BRET assay for interaction of FOXP2 with CtBP1. HEK293 cells were transfected with luciferase-FOXP2 (wild-type (WT) or K674R mutant, donor) and YFP-CtBP1 (acceptor). The control is a nuclear-targeted luciferase. Values are mean corrected BRET ratios ± S.E.M. (n = 3).

SUMOylation can affect the function of transcription factors in several ways^31^. To identify potential effects of SUMOylation on FOXP2 function, we assessed if the K674R SUMOylation site mutant displayed any altered properties in cellular assays. The mutant did not exhibit any differences in subcellular localization, retaining a diffuse nuclear distribution (Fig. 5a). To assess differences in protein expression level, we transfected cells with YFP fusions of wild-type FOXP2 or the K674R mutant, and measured fluorescence intensity over time, relative to the fluorescence intensity of co-transfected mCherry. No differences were observed in the ultimate expression level of the wild-type and mutant proteins, or in the time course of induction of expression (Fig. 5b). To test for differences in protein degradation, cycloheximide was added to cells expressing wild-type FOXP2 or the K674R mutant to arrest protein synthesis, and the decrease in FOXP2 protein over time was monitored by western blotting. For both wild-type and mutant FOXP2, the amount of protein had dropped to approximately 25% of starting levels after 6 h incubation with cycloheximide, and no difference in the rate of degradation was observed between the wild-type and mutant proteins (Fig. 5c). Abolishing the major SUMOylation site therefore does not have a substantial effect on FOXP2 turnover in cultured cells. It is possible that differences in stability might be evident after longer incubations with cycloheximide, but reliable quantification of FOXP2 is precluded by the low levels of remaining protein.

To determine if SUMOylation might affect the transcriptional regulatory activity of FOXP2, we employed a luciferase reporter assay in which luciferase expression is driven by the SV40 viral promoter^3^. As previously reported, wild-type FOXP2 repressed luciferase activity by around 60%, whereas the mutant FOXP2 found in the KE family (R553H), which is unable to bind DNA, did not repress luciferase activity (Fig. 5d)^3^. The K674R mutant did not differ significantly in its repressive capability from the wild-type protein (Fig. 5d). A luciferase assay was also performed using the human SRPX2 promoter. It has previously been reported that FOXP2 represses transcription from this promoter^43^. In our assay, wild-type FOXP2 substantially repressed luciferase activity, and the R553H mutant showed loss of repression, but the K674R mutant again did not differ significantly in its repressive capability from the wild-type protein (Fig. 5d). Thus the loss of the SUMOylation site does not have a generalized effect on the repressive capability of FOXP2.

We then looked to see if abolishing the SUMOylation site affects the ability of FOXP2 to form homodimers^30^. The mutant displayed normal dimerization ability in a BRET assay (Fig. 5e), as might be expected given that FOXP2 dimerization is mediated by the leucine zipper domain, which is not located near to the SUMOylation site (Fig. 4a)^29^,^30^. Finally we employed a BRET assay to assess if the K674R mutant differed in its ability to bind to the co-repressor CtBP1^29^,^30^. Again, no differences were observed between the wild-type and mutant proteins (Fig. 5f). Thus, in our cell-based assays, abolishing the major SUMOylation site in FOXP2 does not have a substantial impact on the behaviour of the protein.

### A FOXP2 mutant that causes speech/language disorder shows reduced SUMOylation

**Figure 6:**
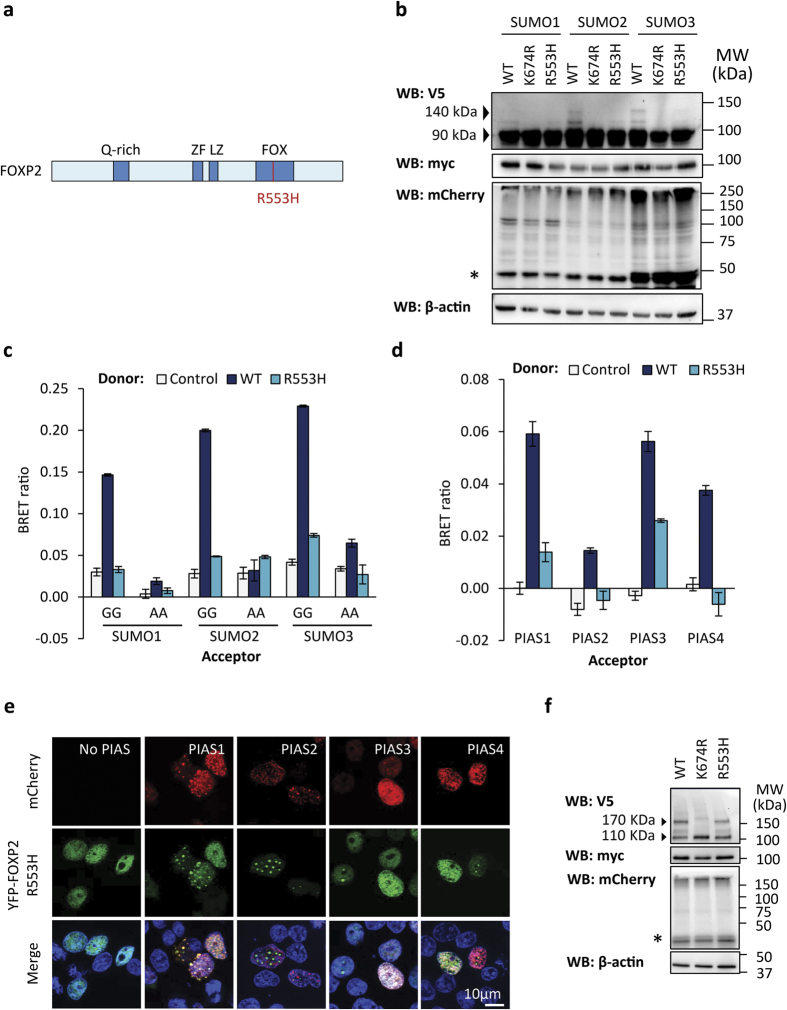
The R553H FOXP2 mutant which causes speech/language disorder exhibits reduced SUMOylation. (**a**) Schematic representation of the FOXP2 R553H mutant. Known domains are shown in dark blue: glutamine-rich region (Q-rich); zinc finger (ZF); leucine zipper (LZ); forkhead domain (FOX). (**b**) Gel shift assay for FOXP2 SUMOylation. HEK293 cells were transfected with V5-tagged FOXP2 (wild-type (WT), K674R or R553H mutant) together with mCherry-SUMO and myc-tagged PIAS1. Top panel: western blot probed with anti-V5 antibody. The 90 kDa species is unmodified FOXP2. The 140 kDa species is FOXP2 modified with mCherry-SUMO. Second panel: western blot probed with anti-myc tag antibody to detect PIAS1. Third panel: western blot probed with anti-mCherry. The asterisk indicates unconjugated mCherry-SUMO. Higher molecular weight species are cellular proteins modified with mCherry-SUMO. Bottom panel: western blot probed with anti-β-actin to confirm equal loading. (**c**) BRET assay for interaction of FOXP2 with SUMO. HEK293 cells were transfected with luciferase-FOXP2 (wild-type (WT) or R553H mutant, donor) and YFP-SUMO (wild-type (GG) or alanine mutant (AA), acceptor). The control donor protein is a nuclear-targeted luciferase. Values are mean corrected BRET ratios ± S.E.M. (n = 3). (**d**) BRET assay for interaction of FOXP2 with PIAS proteins. HEK293 cells were transfected with luciferase-FOXP2 (wild-type (WT) or R553H mutant, donor) and YFP-PIAS (acceptor). The control donor protein is a nuclear-targeted luciferase. Values are mean corrected BRET ratios ± S.E.M. (n = 3). (**e**) Fluorescence micrographs of HEK293 cells transfected with mCherry-PIAS (red) and YFP-FOXP2 R553H mutant (green). Nuclei were stained with Hoechst 33342 (blue). (**f**) Gel shift assay for FOXP2 SUMOylation. HEK293 cells were transfected with FOXP2-UBC9 (wild-type (WT), K674R or R553H mutant) together with mCherry-SUMO3 and myc-tagged PIAS1. Top panel: western blot probed with anti-V5. The 110 kDa species is unmodifed FOXP2-UBC9. The 170 kDa species is FOXP2-UBC9 modified with mCherry-SUMO3. Second panel: western blot probed with anti-myc tag antibody to detect PIAS1. Third panel: western blot probed with anti-mCherry. The asterisk indicates unconjugated mCherry-SUMO. Higher molecular weight species are cellular proteins modified with mCherry-SUMO. Bottom panel: western blot probed with anti-β-actin to confirm equal loading.

Members of the KE family who are affected by speech and language disorder all carry a heterozygous mutation, R553H, within the DNA-binding domain of FOXP2 (Fig. 6a)^1^. This mutation affects a critical residue within the DNA-recognition helix of the FOX domain and abolishes DNA binding^1^,^2^,^3^,^44^. Unexpectedly, a gel shift assay in which cells were transfected with wild-type or mutant FOXP2, together with PIAS1 and SUMO, showed a clear reduction in SUMOylation of the R553H mutant, though it was still modified to a greater extent than the K674R mutant (Fig. 6b). A BRET assay also showed a near total loss of interaction between SUMOs and the R553H mutant in comparison to wild-type FOXP2 (Fig. 6c), in contrast to the partially retained interaction between SUMOs and the K674R mutant (Fig. 4d).

Given that the major SUMOylation site is intact in the R553H mutant, the reduction in SUMOylation may be a consequence of reduced interaction between the mutant and components of the SUMOylation machinery, such as PIASs or UBC9. Consistent with this possibility, the R553H mutant showed reduced or abolished interaction with PIASs in a BRET assay (Fig. 6d). The R553H mutant continued to show some co-localization with PIASs in transfected cells, however the tendency of this mutant to form aggregates makes it unclear if the punctae containing both proteins are nuclear speckles or protein aggregates (Fig. 6e)^3^. Decreased interaction with PIAS1 could account for the relatively higher level of R553H SUMOylation in the gel shift assay compared to the BRET assay, because the overexpression of PIAS1 in the gel shift assay might have a compensatory effect on SUMOylation. Interestingly, the R553H mutant was SUMOylated to a similar extent as wild-type FOXP2 when these proteins were fused to UBC9 (Fig. 6f), suggesting that fusion to UBC9 might rescue a loss of interaction with the SUMOylation machinery in the R553H mutant.

Thus in contrast to the K674R mutant, which is able to interact with PIASs but cannot be SUMOylated, the R553H mutant has reduced interaction with PIASs, but can still be SUMOylated by employing overexpression of PIAS or fusion to UBC9. It is unexpected that the R553H mutation should reduce interaction with PIASs, because truncated forms of FOXP2 that lack the entire FOX domain, and are thus also unable to bind DNA, are still able to interact with PIAS1 (Fig. 1d). The partial mislocalization and increased propensity for protein aggregation resulting from the R553H mutation may contribute to the reduction in interaction, although the majority of mutant protein still displays a normal diffuse nuclear localization, and retains the ability to interact with wild-type FOXP2, indicating that the mutation does not cause gross misfolding of the entire population of molecules^3^,^29^. Potentially the R553H mutation causes a conformational change that blocks the PIAS binding site. Alternatively, the loss of DNA-binding capacity and/or destabilization of the FOX domain resulting from the R553H mutation may permit interactions with other cellular proteins, such as those involved in protein degradation, that in turn interfere with PIAS binding. Although the mechanism by which the R553H mutation reduces interaction with PIAS is unclear, it seems likely that a functional FOX domain is needed in addition to the K674 SUMOylation site to permit normal levels of FOXP2 SUMOylation in cells.

### SUMOylation of other FOXP proteins

**Figure 7:**
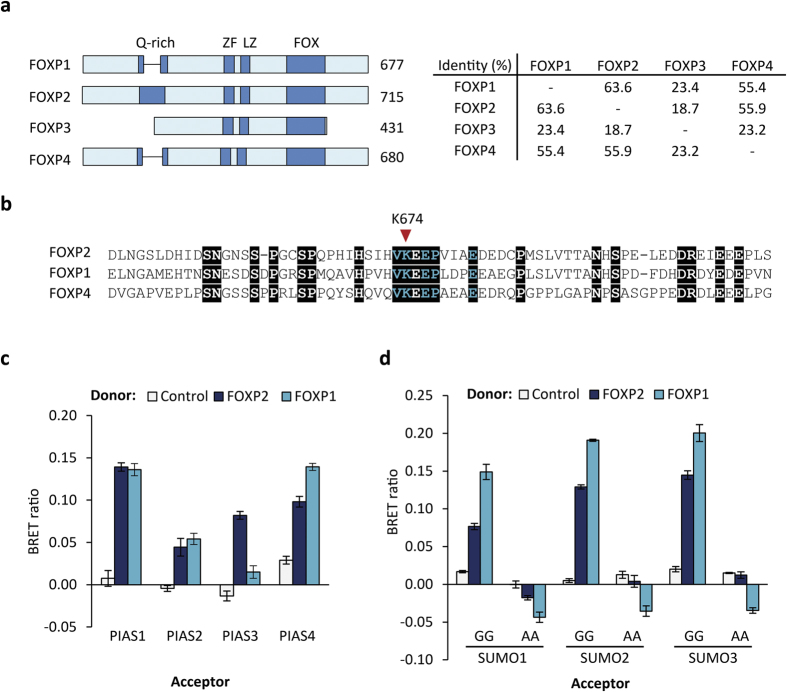
The FOXP2 paralogue FOXP1 is also SUMOylated (**a**) Left: Schematic representation of the FOXP family of proteins. Known domains are shown in dark blue: glutamine-rich region (Q-rich); zinc finger (ZF); leucine zipper (LZ); forkhead domain (FOX). The number of amino acid residues is indicated to the right of the schematic. Right: identity matrix for FOXP proteins. (**b**) Sequence alignment of the region surrounding the SUMOylation site in FOXP proteins. Conserved residues are shown on a black background. Critical residues of the KEPE-type SUMOylation site motif are shown in turquoise and the SUMO conjugation site is labeled. UniProt accession numbers: FOXP2 O15409; FOXP1 Q9H334; FOXP4 Q8IVH2. (**c**) BRET assay for interaction of FOXP1 and FOXP2 with PIAS proteins. HEK293 cells were transfected with luciferase-FOXP1 or luciferase-FOXP2 (donor) and YFP-PIAS (acceptor). The control donor protein is a nuclear-targeted luciferase. Values are mean corrected BRET ratios ± S.E.M. (n = 3). (**d**) BRET assay for interaction of FOXP1 and FOXP2 with SUMO. HEK293 cells were transfected with luciferase-FOXP1 or luciferase-FOXP2 (donor) and YFP-SUMO (wild-type (GG) or alanine mutant (AA), acceptor). The control donor protein is a nuclear-targeted luciferase. Values are mean corrected BRET ratios ± S.E.M. (n = 3).

FOXP2 has three mammalian paralogues, FOXP1, FOXP3 and FOXP4 (Fig. 7a). FOXP1, FOXP2 and FOXP4 exhibit 55–65% sequence identity, are able to form heterodimers, and are expressed in overlapping cell populations in the brain and other organs, suggesting that they may co-operate in the regulation of certain subsets of target genes^10^,^30^,^45^. FOXP3 is structurally divergent, and its expression is restricted to regulatory T lymphocytes^46^. The critical residues of the KEPE SUMOylation motif in FOXP2 are conserved in FOXP1 and FOXP4, which is particularly striking because the C-terminal regions of these proteins generally exhibit a low level of similarity (the region is absent in FOXP3) (Fig. 7a,b). This low level of sequence conservation in the C-terminal region of the FOXP proteins is consistent with the polypeptide in this region being structurally disordered, in order to maintain accessibility of the SUMOylation site^40^.

The conservation of the SUMOylation site in FOXP1 prompted us to assess if this protein may also be subject to PIAS-mediated SUMOylation. The essential role of FOXP1 in brain development has recently come to light since it was discovered that haploinsufficiency of *FOXP1* is associated with intellectual disability, autistic features, expressive speech deficits and dysmorphic features^27^,^47^,^48^,^49^,^50^. The FOXP1-related disorder is more severe than that resulting from haploinsufficiency of *FOXP2*, indicating that the two proteins have non-redundant functions in human brain development^51^. Furthermore, and in contrast to aetiological *FOXP2* variants, all aetiological *FOXP1* variants reported to date have occurred de novo^27^,^47^. In a BRET assay, FOXP1 and FOXP2 exhibited similar levels of interaction with PIAS1, PIAS2 and PIAS4, but unlike FOXP2, FOXP1 showed little or no interaction with PIAS3 (Fig. 7c). BRET assays also showed clear interaction of FOXP1 with all three SUMOs (Fig. 7d). It is therefore likely that FOXP1 is also subject to modification by SUMO1, SUMO2 and SUMO3, at an equivalent site to that in FOXP2 (K636), but that the members of the PIAS family have differing levels of importance in the SUMOylation of FOXP1 and FOXP2. Three proteome-wide studies of SUMOylation in human cell lines have also identified FOXP1 and FOXP4 as substrates for SUMOylation, supporting a conserved role for SUMOylation in the regulation of FOXP transcription factors^52^,^53^,^54^.

## Discussion

In this study we have shown that FOXP2 has a single major SUMOylation site at K674, which can be modified by SUMO1, SUMO2 and SUMO3. This site is fully conserved in orthologues of FOXP2, and lies within the C-terminal region of the protein, which previously had no known function. We have demonstrated that FOXP2 interacts with members of the PIAS family of E3 SUMO ligases, and that this interaction probably involves the SP-RING domain of PIAS and the N-terminal region of FOXP2. The interaction between FOXP2 and PIAS proteins causes relocalization of FOXP2 to nuclear speckles, and promotes SUMOylation of FOXP2. The FOXP2 paralogues FOXP1 and FOXP4 probably also undergo PIAS-mediated SUMOylation at equivalent sites.

SUMOylation is believed to occur in all cell types and across all developmental stages, and thousands of nuclear proteins are thought to be modified in this way^31^. The essential role of SUMOylation in development is evidenced by the early embryonic lethality resulting from *Ubc9* knockout in mice^55^. Global changes in SUMOylation levels during brain development have been documented in mouse and rat, but the functional impact of these changes is uncertain^56^,^57^. Effects of SUMOylation on several neural proteins have been reported, with impacts on neuronal specification and dendritic and synaptic morphogenesis, but for most proteins the function of SUMOylation remains unclear^31^,^58^.

In our cellular assays, abolishing the major SUMOylation site in FOXP2 did not produce changes in subcellular localization, stability, transcriptional regulation, dimerization with wild-type FOXP2, or interaction with the co-repressor CtBP1. Abolition of the SUMOylation site may have effects on unknown protein interactions, or on regulation of a particular subset of target genes. It would therefore be of interest in future to perform proteomic and transcriptomic studies to search for protein-protein interactions and target genes that are affected by loss of the FOXP2 SUMOylation site. At the same time, the failure to observe differences between wild-type FOXP2 and the K647R mutant is not necessarily surprising given the small proportion of wild-type protein molecules modified by SUMOylation at any one time. Like several other post-translational modifications, SUMOylation is a dynamic, reversible process which allows protein activity to be regulated on short time-scales in response to external signals that may change over the course of development. The consequences of loss of SUMOylation in FOXP2 may therefore only be apparent in the context of a developing organism, and SUMOylation of FOXP2 is potentially essential *in vivo* but not in cultured cells. It would therefore be interesting to abolish the FOXP2 SUMOylation site in an animal model, in order to assess the effect of loss of SUMOylation on developmental regulation of gene expression. There are still very few examples of animal models in which a SUMOylation site in a specific protein has been ablated. However, abolishing the SUMOylation sites of the transcription factor NR5A1 in the mouse resulted in aberrant regulation of target genes and prominent endocrine abnormalities, without affecting protein stability or localization^59^, consistent with the suggestion that the consequences of loss of SUMOylation may only be apparent in an organismal context. To our knowledge there has been no systematic survey of rare variants in human developmental disorders for changes likely to affect SUMOylation. Thus, the importance of SUMOylation of specific proteins to normal development is not yet fully appreciated.

The rapid and dynamic nature of SUMOylation makes it well suited as a mechanism for modifying the activity of proteins such as FOXP2 in response to activity within neural circuits. Such mechanisms may be important for supporting neural plasticity, a process in which FOXP2 orthologues have been shown to play a role in animal models^9^,^14^,^21^. The absolute conservation of the SUMOylation site and surrounding KEPE motif in FOXP2 orthologues and in the paralogues FOXP1 and FOXP4 suggests that SUMOylation may be an evolutionarily ancient and conserved mechanism for regulating the activity of these transcription factors in the brain and elsewhere, both during development and in the adult organism.

The selection of target proteins for SUMOylation is mediated by SUMO E3 ligases, such as members of the PIAS family. Our results indicate that multiple PIAS proteins may participate in FOXP SUMOylation *in vivo*, and that different PIASs may be involved in the SUMOylation of different FOXPs. RNA expression data from the Human Protein Atlas (www.proteinatlas.org) suggests that the four PIAS proteins have ubiquitous expression, with PIAS1 and PIAS3 showing moderate expression levels across all tissues tested, whereas PIAS2 and PIAS4 have lower average expression levels with high expression in testis. However the PIAS proteins are not functionally redundant, because *Pias1* knockout mice exhibit perinatal lethality, whereas *Pias2* and *Pias4* knockouts have no obvious phenotype (*Pias3* knockouts have not yet been reported)^60^,^61^,^62^. There may be some temporal or spatial specificity in the interaction of PIASs with FOXPs, and we also do not exclude the involvement of other kinds of SUMO E3 ligase in the SUMOylation of FOXPs.

The reduced level of SUMOylation of the R553H mutant, and the reduced interaction of the mutant with PIAS proteins, indicate that mutations within the FOX domain of FOXP2 can interfere with PIAS-mediated SUMOylation. Interestingly, PIAS1-mediated SUMOylation has also been reported for FOXL2 and FOXA2, which belong to different subfamilies of the FOX transcription factor family^63^,^64^. The different subfamilies of FOX proteins show little similarity outside the FOX domain, raising the possibility that the FOX domain may act in concert with other subfamily-specific protein regions to promote SUMOylation of FOX transcription factors. Aetiological mutations have been reported in the FOX domains of several other FOX transcription factors^65^, but the effects of these mutations on protein SUMOylation have not been investigated. We predict that disorder-related FOX domain mutations would disrupt SUMOylation of other FOX transcription factors, including FOXP1.

Concurrently with the submission of this manuscript, SUMOylation of FOXP2 by SUMO1 and SUMO3 was reported by an independent research group^66^. In agreement with our findings, this report identified K674 as the major SUMOylation site in FOXP2, and found that the R553H variant displays reduced SUMOylation. Furthermore, no effect on protein stability or localization was observed when mutating the SUMOylation site, in line with our data. Some potential small effects on transcriptional regulation in luciferase reporter assays were observed, including for SRPX2, using a similar reporter construct to that employed in our experiments, emphasizing that the effects of SUMOylation on FOXP2-mediated transcriptional regulation warrant further investigation, ideally in a more biologically-relevant model, and that the effects of SUMOylation may be promoter-dependent.

SUMOylation is currently the only confirmed post-translational modification of FOXP2. A key part of the way SUMOylation affects protein function is through interaction with other post-translational modifications, for example by competition with ubiquitination and acetylation for modification of specific lysine side chains. Our findings thus emphasize the need to investigate further the post-translational modifications of FOXP2 in order to understand how the activity of this transcription factor may be dynamically regulated in the developing and adult brain.

## Methods

### Yeast two-hybrid assay

The yeast two-hybrid assay was performed by Dualsystems Biotech AG (Switzerland). The bait construct was produced by fusing the coding sequence of full-length human FOXP2 (Uniprot accession O15409) to the DNA-binding domain of the bacterial transcription factor LexA. Preys consisted of a human foetal brain cDNA library fused to the activation domain of yeast Gal4. LacZ was used as the reporter gene and interactions identified by the presence of blue colour. It was confirmed that transfection of the FOXP2 bait construct alone did not activate transcription of the reporter gene. False positive interactors were removed using the bait dependency test to identify prey constructs which activated transcription without co-transfection with the FOXP2 bait construct.

### DNA constructs

The coding sequences of PIAS1 (NM_016166.1), PIAS2 (NM_004671.3), PIAS3 (NM_006099.3), PIAS4 (NM_015897.1), SUMO1 (NM_003352.4), SUMO2 (NM_006937.3), and SUMO3 (NM_001286416.1), and a 1146 bp region of the promoter of SRPX2, were amplified from human foetal brain cDNA using the primers listed in Supplementary Table S4. The cloning of wild-type FOXP2, FOXP1 and CtBP1, and of synthetic truncated forms of FOXP2, has been described previously^27^,^28^,^29^. For expression of fusion proteins with *Renilla* luciferase, YFP and mCherry, cDNAs were subcloned into the pLuc, pYFP and pmCherry expression vectors, respectively, which have been described previously^28^,^29^. For expression of proteins with three tandem N-terminal Myc tags or an N-terminal V5 tag, cDNAs were subcloned into vectors which were created by modification of the vector pEGFP-C2 (Clontech), and have an identical backbone to the pLuc, pYFP and pmCherry vectors, with the exception of the N-terminal tag and polylinker. To generate the FOXP2-UBC9 fusion protein, the UBC9 coding sequence (NM_194260.2) plus 58 upstream nucleotides were fused to the 3′ end of the FOXP2 coding sequence in the V5-tag vector, removing the FOXP2 stop codon. The FOXP2 K674R and R553H mutants, PIAS1 C350S mutant, and SUMO alanine mutants were generated by site-directed mutagenesis using the Quick-Change Site-Directed Mutagenesis kit (Stratagene) following the manufacturer’s protocol. Primers used in site-directed mutagenesis are listed in Supplementary Table S5. The SRPX2 luciferase reporter plasmid was generated by subcloning a 1146 bp region of the SRPX2 promoter into the promoterless firefly luciferase vector pGL4.23 (Promega). All constructs were verified by Sanger sequencing. Plasmid sequences are available upon request.

### Cell culture and transfection

HEK293 were obtained from ECACC (cat. no. 85120602) and cultured in DMEM supplemented with 10% foetal bovine serum. HeLa-SUMO3 cells, which stably express SUMO3 with an N-terminal hexahistidine tag^42^, and the parental HeLa cell line, were kindly provided by Professor Ronald Hay and Dr Michael Tatham, and were cultured in DMEM supplemented with 10% foetal bovine serum (with the addition of 5 μM puromycin for the HeLa-SUMO3 cell line). Transfections were performed using GeneJuice (Merck-Millipore) according to the manufacturer’s instructions.

### BRET assay

BRET assays were performed as described^29^.

### Fluorescence microscopy

HEK293 cells were seeded on coverslips coated with poly-L-lysine. Cells were cultured for 30 h post-transfection, and then fixed with methanol and nuclei were stained with Hoechst 33342. Fluorescence images were acquired using a Zeiss LSM510 confocal microscope with LSM Image Software or a Zeiss Axio Imager 2 upright fluorescence microscope with ApoTome.2 using ZEN Image software.

### Gel shift assay

HEK293 cells were transfected in 6-well plates and cultured for 48 h. Cells were lysed in 300 μL of Laemmli sample buffer containing 10% Tris(2-carboxyethyl)phosphine hydrochloride (TCEP) and incubated for 10 min at 95 °C. Proteins were resolved on 10% SDS-polyacrylamide gels and transferred to PVDF membranes using a TransBlot Turbo Blotting apparatus (Bio-Rad). Membranes were blocked in PBS containing 5% milk and 0.1% Tween-20 and incubated overnight at 4 °C with primary antibody. The following antibodies were used: anti-GFP (Clontech cat. no. 632380, 1:8000, for YFP constructs); anti-mCherry (Novus cat. no. NBP1-96751, 1:1000); anti-V5 tag (Genetex cat. no. GTX42525, 1:3000); anti-Myc tag (Abcam cat. no. ab9106, 1:1000); anti-β-actin (Sigma cat. no. A5441, 1:10,000). After washing, membranes were incubated with horseradish peroxidase-conjugated goat anti-mouse or anti-rabbit IgG for 45 min at room temperature. Proteins were visualized using Novex ECL Chemiluminescent Substrate Reagent Kit (Life Technologies) and a ChemiDoc XRS + imaging system (Bio-Rad). Densitometry was performed using the Chemidoc XRS + System image analysis software (Bio-Rad).

### Pull-down assay

Purification of SUMO3 conjugates from HeLa cells stably expressing His-tagged SUMO3 was performed as previously described^42^. Briefly, cells were seeded in 6-well plates and transfected with YFP-tagged FOXP2 variants or YFP alone. After 48 h cells were lysed in 6 M Guanidinium-HCl, 10 mM Tris, 100 mM Sodium phosphate buffer pH 8.0, 5 mM β-mercaptoethanol and 5 mM imidazole. An aliquot of the lysate (10%) was retained as the input sample and the remainder was incubated with His-tag Dynabeads (Life Technologies) overnight at 4 °C with rotation. Beads were washed with 8 M Urea, 10 mM Tris pH 6.3, 100 mM sodium phosphate buffer, 0.1% Triton x-1000 and 5 mM β-mercaptoethanol. SUMO3 conjugates were eluted by incubation at room temperature for 20 min in 200 mM imidazole, 150 mM Tris pH 8.0, 5% SDS, 30% glycerol, 720 mM β-mercaptoethanol and 0.0025% bromophenol blue. Western blotting was performed as described above; His-tagged SUMO3 conjugates were detected using an anti-His tag antibody (Abgent cat. no. AM1010a, 1:1000).

### Protein degradation assay

HEK293 cells were transfected in 6-well plates and cultured for 48 h. Cycloheximide was added for the indicated times at a final concentration of 50 μg/ml. Cells were lysed for 10 min at 4 °C with 100 mM Tris pH 7.5, 150 mM NaCl, 10 mM EDTA, 0.2% Triton X-100, 1% PMSF, and protease inhibitor cocktail. Cell lysates were cleared by centrifugation at 10,000×*g* for 3 min at 4 °C. Gel electrophoresis, western blotting and densitometry were performed as described above.

### Fluorescence-based measurement of protein expression levels

HEK293 cells were transfected with YFP-FOXP2 and mCherry in clear-bottomed black 96-well plates in triplicate. Cells were cultured in a TECAN M200PRO microplate reader at 37 °C with 5% CO_2_. Fluorescence intensity measurements were taken at multiple time points. For each well and time point, the background-subtracted YFP intensity was divided by the background-subtracted mCherry intensity. Triplicate conditions were averaged.

### Luciferase assays

HEK293 cells were seeded in clear-bottomed white 96-well plates and transfected in triplicate. For the SV40 assay, cells were transfected with 12 ng of pGL3-promoter firefly luciferase reporter construct (Promega), 5 ng of pRL-TK *Renilla* luciferase normalization control (Promega), and 16 ng of YFP-FOXP2 (wild-type or K674R or R553H mutant) or YFP control construct. For the SRPX2 assay, cells were transfected with 4.3 ng of SRPX2 luciferase reporter construct, 5 ng of pGL4.74 *Renilla* luciferase normalization control (Promega), and 45 ng of YFP-FOXP2 (wild-type or K674R or R553H mutant) or YFP control construct. After 48 h, luciferase activity was measured in a TECAN F200PRO microplate reader using the Dual-Luciferase Reporter Assay system (Promega).

## Additional Information

**How to cite this article**: Estruch, S. B. *et al*. The language-related transcription factor FOXP2 is post-translationally modified with small ubiquitin-like modifiers. *Sci. Rep*. **6**, 20911; doi: 10.1038/srep20911 (2016).

## Supplementary Material

Supplementary Information
