# Supplementary material for: The language-related transcription factor FOXP2 is post-translationally modified with small ubiquitin-like modifiers
Source: Sci Rep. 2016 Feb 12;6:20911. doi: 10.1038/srep20911 (PMC4751435; doi:10.1038/srep20911)
Supplement: Supplementary Information [file srep20911-s1.pdf]

The language-related transcription factor FOXP2 is post-translationally modified with small ubiquitin-like modifiers

Sara B. Estruch, Sarah A. Graham, Pelagia Deriziotis, Simon E. Fisher.

Supplementary Table S1: Summary of yeast two-hybrid results

DNA was isolated from positive colonies, the cDNAs of the prey constructs were sequenced, and BLAST search was used to identify the proteins encoded by positive clones. Proteins represented by two or more clones are listed.

**HGNC symbol**	**Number of preys**
PIAS1	7
CHD3	4
FXYD6	4
NREP	3
FKBP1A	2
NRGN	2

Supplementary Table S2: Prediction of putative FOXP2 SUMOylation sites

The table lists all the lysine residues in FOXP2 (Uniprot O15409). SUMOylation sites were predicted using three web-based algorithms: Joined Advanced SUMOylation Site and SIM Analyser (JASSA, www.jassa.fr/), SUMOplot analysis Program (www.abgent.com/SUMOplot), and GPS-SUMO (SUMOsp.biocuckoo.org/). JASSA uses a scoring system based on a Position Frequency Matrix derived from the alignment of experimental SUMOylation sites. One of the two sites identified by JASSA corresponds to an inverted consensus SUMOylation site D/E-X-K-Ψ, where Ψ = A/F/I/L/M/P/V/W; X = any amino acid. The second is a standard consensus SUMOylation site in the context of a negatively charged amino acid-dependent SUMOylation motif (NDSM): Ψ-K-X-D/E-X-α_6, where 2 out of 6 α must be D/E. SUMOplot predicts the probability (0-1) for the major SUMO canonical consensus sequence Ψ-K-X-D/E where Ψ =I/L/V. The GPS-SUMO score is based on 983 SUMOylation sites in 545 proteins collected from the literature.

Residue	Sequence context	JASSA	SUMOplot	GPS-SUMO
K74	LLLQQQTSGLKSPKSSDKQRP		0.80	
K77	QQQTSGLKSPKSSDKQRPLQV			
K81	SGLKSPKSSDKQRPLQVPVSV			
K140	LQQQQLQEFYKKQQEQLHLQL			
K141	QQQQLQEFYKKQQEQLHLQLL			
K195	QQQQQQQHPGKQAKEQQQQQQ			
K198	QQQQHPGKQAKEQQQQQQQQQ			
K271	LSPAEIQQLWKEVTGVHSMED			
K285	GVHSMEDNGIKHGGLDLTTNN		0.77	
K306	SSSTTSSNTSKASPPITHHSI			
K349	SHTLYGHGVCKWPGCESICED			
K365	SICEDFGQFLKHLNNEHALDD			
K397	VVQQLEIQLSKERERLQAMMT			
K417	THLHMRPSEPKPSPKPLNLVS	High (Inverted)		
K421	MRPSEPKPSPKPLNLVSSVTM			
K433	LNLVSSVTMSKNMLETSPQSL			
K482	VGAIRRRHSDKYNIPMSSEIA			
K499	SEIAPNYEFYKNADVRPPFTY			
K549	AYFRRNAATWKNAVRHNLSLH			
K560	NAVRHNLSLHKCFVRVENVKG		0.17	
K569	HKCFVRVENVKGAVWTVDEVE			
K582	VWTVDEVEYQKRRSQKITGSP			
K587	EVEYQKRRSQKITGSPTLVKN			
K596	QKITGSPTLVKNIPTSLGYGA			
K674	PQPHIHSIHVKEEPVIAEDED	High (NDSM)	0.93	24.943

Supplementary Table S3: Prediction of putative FOXP2 SUMO-interacting motifs (SIMs)

SUMO-interacting motifs (SIMs) were predicted using two web-based algorithms: Joined Advanced SUMOylation Site and SIM Analyser (JASSA, www.jassa.fr/), and GPS-SUMO (SUMOsp.biocuckoo.org/). JASSA uses a scoring system based on a Position Frequency Matrix derived from the alignment of experimental SIMs and the maximum predictive score is 38. The GPS-SUMO score is based on 151 SIMs in 80 proteins collected from the literature. In both cases, putative SIMs were only identifiable by using the lowest possible detection threshold. Potential SIMs are indicated in bold type.

Residues	Sequence context	JASSA	GPS-SUMO
47-50	DTSSEVST**VELL**HLQQQQAL	0.275	
49-52	SSEVSTVE**LLHL**QQQQALQA	0	
63-66	QQALQAAR**QLLL**QQQTSGLK	0	
148-151	KKQQEQLH**LQLL**QQQQQQQQ	0.053	
233-236	QQLQQQQH**LLSL**QRQGLISI	0.018	
241-244	LLSLQRQG**LISIP**PGQAALP	0.097	28.42
423-426	EPKPSPKP**LNLV**SSVTMSKN	0.01	
618-624	QAALA**ESSLPLL**SNPGLINN	0.036	

Supplementary Table S4: Cloning primer sequences
Restriction sites are underlined.

Gene	Forward primer	Site	Reverse primer	Site
PIAS1	AAGATCTCGGCGGACAGTGCGGAACTAAAGC	BglII	CGCTAGCTTAGTCCAATGAAATAATGTCTGGT	NheI
PIAS2	GGATCCTGGCGGATTTCGAAGAGTTG	BamHI	TCTAGATTAGTCCAATGAGATGATGTCAGG	XbaI
PIAS3	AGATCTTGGCGGAGCTGGGCGAATTAAA	BglII	TCTAGATCAGTCCAGGGAAATGATGTC	XbaI
PIAS4	GGATCCTGGCGGCGGAGCTGGTG	BamHI	TCTAGATCAGCAGGCCGGCACCAGGCCCTT	XbaI
SUMO1	GGATCCTGTCTGACCAGGAGGCAAAACCTT	BamHI	TCTAGACTAAACTGTTGAATGACCCCC	XbaI
SUMO2	GGATCCTGGCCGACGAAAAGCCCAAGGAAG	BamHI	TCTAGATTAGTAGACACCTCCCGTCTG	XbaI
SUMO3	GGATCCTCTCCGAGGAGAAGCCCAAGGAG	BamHI	TCTAGACTAGAAACTGTGCCCTGCCAG	XbaI
UBC9	AGATCTGGAACACCTGTCCGCTACGCTC	BglII	TCTAGATTATGAGGGCGCAAACTTCTT	XbaI
SRPX2 promoter	GGTACCCTCTGCCTCCTGGGTTCAAG	KpnI	AAGCTTGATGGGGGAGAAGGAACACA	HindIII

Supplementary Table S5: Site-directed mutagenesis primer sequences

Mutant	Primer 1	Primer 2
FOXP2 K674R	CATACATTCAATCCACGTCAGGGAAGAGCCAGTGATTG	CAATCACTGGCTCTTCCCTGACGTGGATTGAATGTATG
PIAS1 C350S	ACATTGTAGATGAGAACTTGTAAGGGCCCGACAC	GTGTCGGGCCCTTACAAGTTCTCATCTACAATGT
SUMO1 AA	AGACTAAACTGTTGAATGAGCCGCCGTTTGTTCCTGATAAAC	GTTTATCAGGAACAAACGGCGGCTCATTCAACAGTTTAGTCT
SUMO2 AA	TTCTAGATCAGTAGACAGCTGCCGTCTGCTGTTGGAAC	GTTCCAACAGCAGACGGCAGCTGTCTACTGATCTAGAA
SUMO3 AA	CTCTCCGGCACAGCTGCCGTCTGCTGCT	AGCAGCAGACGGCAGCTGTGCCGGAGAG
